# HIV Transmission in Correctional Facility

**DOI:** 10.3201/eid1204.050484

**Published:** 2006-04

**Authors:** Abe Macher, Deborah Kibble, David Wheeler

**Affiliations:** * US Public Health Service (retired), Bethesda, Maryland, USA;; † Metropolitan Washington Council of Governments, Washington, DC, USA;; ‡ Infectious Diseases Physicians, Annandale, Virginia, USA

**Keywords:** inmate, jail, receptive anal sex, HIV, acute retroviral syndrome

## Abstract

Acute retroviral syndrome developed in an inmate in a detention center after he had intercourse with 2 HIV-infected inmates. Correctional facilities house a disproportionate number of HIV-infected persons, and most do not provide inmates with condoms. Correctional healthcare providers should be familiar with primary HIV infection and acute retroviral syndrome.

Correctional facilities house a disproportionate number of HIV-infected inmates (*1*) and are a setting for unprotected sexual intercourse (*2*). Although symptoms of acute retroviral syndrome develop in up to 89% of persons newly infected with HIV (*3*), the timely recognition and diagnosis of primary HIV infection and initiation of antiretroviral treatment before HIV seroconversion have rarely been reported from a correctional facility.

## The Case

In October 2003, a man with a history of noninjection multiple-drug abuse (including methamphetamine) was admitted to a detention center (regional jail). His pre-incarceration history included unprotected sex with men in the community, most recently in April 2003; however, results of multiple serologic assays for HIV performed in the community had been negative, most recently in June 2003 and December 2002. He had an unremarkable past medical history.

On December 31, 2003, this patient came to the correctional facility's medical clinic with perianal and rectal discomfort; perianal condylomata were present. He reported that during December he had consensual, unprotected, receptive anal intercourse with 2 male inmates at the correctional facility; both of these inmates had chronic HIV infection. One, who was not receiving antiretroviral treatment, had a plasma HIV RNA level of 53,000 copies/mL; the second, who was receiving antiretroviral treatment, had a plasma HIV RNA level of 92 copies/mL. On January 6, enzyme immunoassay (EIA) testing of the index patient for HIV was negative.

On January 9, the patient came to the medical clinic with fever, sore throat, myalgia, headache, vertigo, nausea, and vomiting. His oral temperature was 40.7°C, and he was profusely diaphoretic. Posterior pharyngeal erythema was present, as well as tender, minimally enlarged, anterior cervical lymphadenopathy. Laboratory testing showed plasma HIV RNA of 436,000 copies/mL; CD4+ T-lymphocyte count of 616 cells/μL (28%); serum alkaline phosphatase 183 IU/L; and negative serologic test results for hepatitis A, B, and C viruses. He denied participating in tattooing or injection drug use.

On January 13, he reported vertigo and urinary retention; his temperature was 39.4°C, and he was ataxic. Acute urinary retention required urethral catheterization. On January 14, physical examination showed bilateral horizontal nystagmus and perianal ulcerations; plasma HIV RNA was >750,000 copies/mL. On January 15, a second EIA for HIV was negative; however, a Western blot of that serum sample showed an equivocal p24 band.

On January 16, he reported difficulty defecating and urinating. Physical examination showed a scattered macular exanthem of discrete erythematous macules on the trunk and extremities with involvement of the palms; oral mucositis; a tender prostate; and a friable, mildly inflamed anal mucosa with some ulcerations and excoriations. His persistent urinary retention required short-term urethral catheterization. Swab cultures of the rectum for herpesvirus, *Chlamydia*, and *Neisseria gonorrhoeae* were negative. Serologic testing for syphilis was negative.

On January 20, a CD4+ T-lymphocyte count drawn on January 15 showed 338 cells/μL (26%); plasma HIV RNA level was 234,000 copies/mL. His exanthem had become maculopapular, diffuse, and pruritic. Antiretroviral therapy with efavirenz, zidovudine, and lamivudine was initiated at the correctional facility to treat his primary HIV infection. A genotype of his pretreatment HIV isolate collected on January 14 showed sensitivity to all antiretroviral agents.

On January 21, he complained of paresthesias involving the tips of his fingers. On January 22, he was able to urinate spontaneously. On January 30, he reported a poor appetite; his weight had decreased 1.4 kg since January 16; physical examination showed a resolving, salmon-colored macular exanthem across the trunk and upper and lower extremities and resolution of his oral and anal lesions.

On February 4, his plasma HIV RNA was 2,463 copies/mL, and his CD4+ T-lymphocyte count was 1,575 cells/μL. An EIA for HIV was positive, and a Western blot was positive with bands present for p24, gp40, p55, and gpl60. On February 9, he reported that his appetite had recovered and his paresthesias had resolved; his weight had increased 0.5 kg since January 30. On March 19, his plasma HIV RNA was <50 copies/mL, and his CD4+ T-lymphocyte count was 1,056 cells/μL. On April 1, the court ordered that he be released, and he moved to another state.

## Conclusions

In April 2005, Lambert et al. (*4*) reported concerns that a resurgence of HIV/AIDS may be imminent, fueled in part by increasing indicators of high-risk behavior in the gay and bisexual population. The March 2005 report by Markowitz et al. (*5*) regarding men who have sex with men, use of methamphetamine, and transmission of HIV underscores these concerns. The high prevalence of HIV infection in overcrowded and understaffed correctional facilities further accentuates these concerns and poses a public health challenge.

On December 31, 2002, 2.0% of state prison inmates were positive for HIV (*1*); among interviewed jail inmates, 1.3% disclosed they were HIV positive. Estimates of the proportion of inmates who indulge in homosexual intercourse while in prison range from 2% to 65%, and most of this sexual contact is likely unsafe because few correctional facilities address the issue of intraprison sex or distribute condoms (*2*). Nevertheless, inmate-to-inmate transmission of HIV has rarely been documented. Taylor et al. (*6*) proposed that the paucity of evidence for transmission of HIV infection within correctional facilities is probably accounted for by the difficulties in determining the time of HIV seroconversion in relation to the period of incarceration, rather than by the rarity of the event.

Krebs and Simmons (*2*) used surveillance data from a 22-year period (January 1, 1978–January 1, 2000) to identify inmates who contracted HIV while incarcerated in the Florida state prison system. They reported that a minimum of 33 inmates contracted HIV while in prison, compared to 238 who contracted HIV after leaving prison; inmates were more likely to have contracted HIV in prison by having sex with other men than through injection drug use.

Additional reports of HIV transmission in correctional facilities have been published from Illinois (8 HIV seroconversions) (*7*), Nevada (2 seroconversions) (*8*), Maryland (2 seroconversions) (*9*), Australia (1 seroconversion) (*10*), and Scotland (*11*). Yirrell et al. (*11*) determined that 13 inmates had acquired HIV infection by sharing needles during their incarceration.

Acute retroviral syndrome and primary HIV infection may be frequently unsuspected by the evaluating clinician because the signs and symptoms are relatively nonspecific. However, within correctional facilities, the diagnosis of primary HIV infection should be considered in the differential diagnosis of any inmate with an acute febrile illness associated with pharyngitis and mucocutaneous lesions. Our report is limited in that virus was not sequenced to document transmission between inmates.

Early diagnosis of primary HIV infection can lead to successful antiretroviral intervention (*12*) and prevention of secondary transmission. Whether antiretroviral treatment of acute HIV infection results in long-term virologic, immunologic, or clinical benefit is unknown. In October 2005, the US Department of Health and Human Services Clinical Practices Panel noted that antiretroviral treatment of acute HIV infection is optional. If the clinician and patient elect to treat acute HIV infection with antiretroviral therapy, treatment should be implemented with the goal of suppressing plasma HIV RNA to below detectable levels; resistance testing at baseline will likely optimize virologic response (*13*).

We urge correctional facilities to address the issue of unprotected sex among inmates and the associated transmission of sexually transmitted diseases within institutions (*14*). In 2001, Wolfe et al. (*14*) reported that from 1991 to 1999, >5 outbreaks of syphilis occurred in Alabama prisons; multiple concurrent sex networks involving 4, 7, and 10 inmates were identified in the 1999 outbreak. Wolfe et al. recommended that condom distribution should be used to control sexually transmitted disease in correctional facilities. Nevertheless, in 2006, <1% of US correctional facilities provide inmates with condoms. Reasons for not providing condoms include the conflict with policies forbidding sexual intercourse (or sodomy) and the potential for condoms to be used as weapons or to smuggle contraband (*15*). In contrast, condoms are available to inmates in all Canadian federal prisons and some provincial prisons; few problems related to condom distribution have been reported from those systems (*15*). Wolfe et al. proposed that providing condoms to prisoners may yield additional public health advantages beyond the prison walls if exposure to and experience with condoms in this setting translate into increased use after release.

Correctional staff and inmates should be educated about the consequences of unprotected sex and the signs and symptoms of acute retroviral syndrome. Because many correctional systems contract for medical care, and because staff turnover rates are high, annual education should be implemented. Education for staff who screen sick inmates is critical (*14*), and all inmates should have access to HIV counseling and testing.
